# Human Motor Neuron Progenitor Transplantation Leads to Endogenous Neuronal Sparing in 3 Models of Motor Neuron Loss

**DOI:** 10.4061/2011/207230

**Published:** 2011-05-23

**Authors:** Tanya J. Wyatt, Sharyn L. Rossi, Monica M. Siegenthaler, Jennifer Frame, Rockelle Robles, Gabriel Nistor, Hans S. Keirstead

**Affiliations:** ^1^Department of Anatomy & Neurobiology, Reeve-Irvine Research Center, Sue and Bill Gross Stem Cell Research Center, School of Medicine, 2002 Sue and Bill Gross Hall: A CIRM Institute, University of California at Irvine, Irvine, CA 92697-4292, USA; ^2^California Stem Cell, Inc., 18301 Von Karmen Avenue, Irvine, CA 92612, USA

## Abstract

Motor neuron loss is characteristic of many neurodegenerative disorders and results in rapid loss of muscle control, paralysis, and eventual death in severe cases. In order to investigate the neurotrophic effects of a motor neuron lineage graft, we transplanted human embryonic stem cell-derived motor neuron progenitors (hMNPs) and examined their histopathological effect in three animal models of motor neuron loss. Specifically, we transplanted hMNPs into rodent models of SMA (Δ7SMN), ALS (SOD1 G93A), and spinal cord injury (SCI). The transplanted cells survived and differentiated in all models. In addition, we have also found that hMNPs secrete physiologically active growth factors *in vivo*, including NGF and NT-3, which significantly enhanced the number of spared endogenous neurons in all three animal models. The ability to maintain dying motor neurons by delivering motor neuron-specific neurotrophic support represents a powerful treatment strategy for diseases characterized by motor neuron loss.

## 1. Introduction

Human embryonic stem cells (hESCs) can differentiate into any cell type, are amenable to genetic manipulation, and readily self-renew *in vitro*. Directed differentiation of hESCs into high purity populations of a defined cell type can be used to design effective treatments that are both cell and site specific. Stem cell-based transplantation is an inherently combinatorial therapeutic approach, in that it not only replaces dead or dying cells, but also provides a substrate for endogenous growth, bridges gaps where tissue is lost to injury or disease, and provides neurotrophic support for the endogenous environment. 

Amyotrophic lateral sclerosis (ALS), spinal muscular atrophy (SMA), and spinal cord injury (SCI) are all diseases of motor neuron loss that could potentially benefit from transplantation of hESC-derived cells. ALS is the most common form of motor neuron disease [1] and can be either sporadic or familial [2–5]. Approximately 5–10% of those diagnosed have a positive family history of the disease [1, 6]. Although the etiology of the sporadic form of ALS is largely unknown [5], the familial form has a strong genetic linkage to point mutations in either *TAR DNA-binding protein 43* (TDP-43), *FUS/TLS*, or the *Cu/*Zn superoxide *dismutase 1* (SOD1) genes [7]. The average age of onset is between 40 and 60 years, and the disease is generally fatal within 1–5 years of onset [1, 6]. SMA is also characterized by motor neuron loss and is the leading genetic cause of infantile death [8, 9]. The disease manifests by roughly 6 months of age in the most severe cases (type I), and death follows in ~2 years without supporting intervention [9, 10]. It is caused by a deletion or mutation in the *Survival Motor Neuron *(*SMN1*) gene [9, 11] in approximately 1 of every 10,000 live births [9, 10]. SCI involves several degenerative processes including but not limited to axonal and cellular damage to the motor neuron compartment [12]. Each of these three neurodegenerative diseases is characterized by the progressive loss of muscle control and death of motor neurons within the spinal cord. Because the adult central nervous system (CNS) has a limited ability to repair itself, therapeutic strategies have focused on ameliorating secondary cellular degeneration [12, 13], promoting endogenous repair mechanisms [14–16], and cell replacement [12, 17–19].

There is much evidence that transplantation of stem and progenitor cells into the CNS produces functional improvement even in the absence of functional integration. Human neural stem cells constitutively secrete the neurotrophins NGF, BDNF, and GDNF that promote growth of injured axons following spinal cord injury [14, 20]. At least a portion of the functional recovery seen after transplantation of hESC-derived oligodendrocyte progenitor cells into models of SCI has been attributed to secretion of growth factors by the transplanted cells [12, 20–22]. This data indicates that growth factors secreted by transplanted stem cells can significantly influence pathogenesis of the diseased/injured spinal cord [20].

We have previously documented a method to differentiate hESCs into high purity populations of human embryonic stem cell-derived motor neuron progenitors (hMNPs) [20]. In this study, we sought to investigate whether hMNPs secrete growth factors that have neurotrophic effects on neurons in culture and in three different models of motor neuron loss. We demonstrate that hMNPs secreted physiologically active growth factors *in vitro* and *in vivo* and significantly enhanced the number of endogenous neurons following transplantation in all three animal models of motor neuron loss. From a therapeutic perspective, these observations indicate that cell transplantation benefits pathogenesis by growth factor secretion, in addition to cell replacement.

## 2. Materials and Methods

### 2.1. Ethics Statement

All animal work was carried out in accordance with the UCI Institutional Animal Care and Use Committee (2007-2725). Animals received appropriate postsurgical care including subcutaneous saline, prophylactic Baytril (2.5 mg/kg/d, s.c.; Bayer, Shawnee Mission, KS), and Buprenorphine (0.025 mg/kg/d, s.c.; Western Medical Supply, Los Angeles, CA) for three days. Animals were inspected for weight loss, dehydration, discomfort, and autophagia, with appropriate veterinary care as needed. Research involving human participants was approved by the UCI Institutional Review Board (2008-6467) and the UCI Medical Center with written informed consent. All work involving human embryonic stem cells was approved by the UCI Human Embryonic Stem Cell Research and Oversight Committee (2007-5645).

### 2.2. Derivation of hMNPs from hESCs

hMNPs were derived from hESC lines hCSC14, hCSC14-CL1 (California Stem Cell, Inc., Irvine, CA) at passages 15–17 and H7 at passages 26–38. hESCs were expanded on Matrigel (BD Biosciences, San Jose, CA) for 1–3 weeks in StemBlast media (California Stem Cell, Inc., Irvine, CA) supplemented with 20 ngbFGF/mL (Millipore, Billerica, MA). Cells were then transferred to ultra low-binding dishes (Corning, NY) and suspended in motor neuron (MN) differentiation media, which consisted of osmolarity adapted DMEM : F12 mixture (260 mOsm) supplemented with Glutamax, B27 supplement (Gibco-Invitrogen, Carlsbad, CA), insulin 10 *μ*g/mL, sodium selenite 1 ng/mL, transferrin 10 *μ*g/mL (Sigma Aldrich, St. Louis, MO), MgSO_4_ 0.5 Mm, and bFGF 5 ng/mL (Millipore, Billerica, MA). Cells were exposed to this media for 5 days, supplemented with 10 *μ*M retinoic acid (RA; from 20 mM stock solution in DMSO, Sigma-Aldrich, St. Louis, MO). Cultures were fed daily for the duration of RA treatment, then every 2 days for 16 subsequent days. At day 21, cell clusters were transferred to adherent laminin substrate (20 *μ*g/cm^2^). At day 28, the conditioned media was collected for *in vitro* assays, or the cells were prepared for transplantation. Subsets of cells were plated onto laminin coated 4-well chamber slides (Nunc; Fisher Scientific, Pittsburgh, PA) for immunocytochemical profiling.

### 2.3. Preparation of MN-Astrocyte Cocultures

To assess maturation into a motor neuron phenotype, hMNPs were plated onto primary astrocytes on day 28 and allowed to mature for 3 weeks thereafter according to previously published protocols [20] and Dr. John Weiss of UC, Irvine. Briefly, astrocyte cultures were prepared from cerebral cortices of early postnatal (1–3 day) mice. After removing cortices, the tissue was dissociated and plated at a density of about 2 × 10^4^ cells/cm^2^, in media consisting of Eagle's minimal essential medium (MEM, Earle's salts, supplied glutamine free; serum; Gibco; Grand Island, NY), supplemented with 10% heat-inactivated horse serum (Gibco), 10% fetal bovine serum (Gibco), glutamine (2 mM; Gibco), glucose (total 25 mM), and epidermal growth factor (final concentration 10 ng/mL) in 15 mm Primaria-coated culture plates (Falcon; Franklin Lake, NJ). Cultures were maintained in a 37°C/5% CO_2_ incubator. Under these conditions, there is virtually no neuronal survival, the astrocyte monolayers generally become confluent after ~2 weeks. After confluence, the astrocyte monolayers were seeded with hMNP sat a density of 50,000 cells/cm^2^. Subsequently, media was exchanged twice a week. Mature motor neurons were immunostained and characterized as below.

### 2.4. Immunocytochemical Labeling

Immunocytochemistry was performed using standard protocols, as described previously [20]. The following proteins were evaluated: anti-TUJ1 (1 : 1000), anti-HB9 (1 : 100; MNR2 Ab from DSHB Developmental Studies Hybridoma Bank), anti-Islet1 (1 : 200), anti-GFAP (1 : 500) anti-SMI32 (1 : 1000), and anti-ChAT (1 : 100; Millipore at 4°C overnight). Primary antibody application was followed by fluorescent secondary antibody application (Alexafluor-488 or -594 conjugated; Invitrogen, Carlsbad, CA).

### 2.5. Primary Cortical Cultures

Mixed primary cortical cultures were prepared from postnatal day 1 Sprague-Dawley rats for neurite outgrowth with and without hMNP-conditioned media (CM) or MN differentiation media (without conditioning) as previously described in Rossi et al. [20]. Briefly, the cortex was mechanically dissociated, trypsinized, and spun at 1500 g for 5 minutes. The pellet was resuspended in Neurobasal media (Invitrogen, Carlsbad, CA). 40,000 cells were plated onto poly-L-lysine coated chamber slides for neurite outgrowth [23]. After seeding, the chamber slides were fed for the next 2 days with Neurobasal media supplemented with 10 ng/mL FGF until neuronal attachment.

### 2.6. In Vitro Growth Factor Secretion Assay

To determine whether hMNP-conditioned media had neurotrophic effects on neurite outgrowth, MN differentiation media was conditioned for 48 hours in the presence of hMNPs and removed for presentation to cortical neurons. Primary cortical neurons were fed with either MN differentiation media or hMNP-CM diluted 1 : 1 with MN differentiation media (*n* = 4/group). MN differentiation media (without conditioning) consisted of osmolarity adapted DMEM : F12 mixture (260 mOsm) supplemented with Glutamax, B27 supplement (Gibco-Invitrogen, Carlsbad, CA), insulin 10 mg/mL, sodium selenite 1 ng/mL, transferrin 10 mg/mL (Sigma Aldrich, St. Louis, MO), MgSO_4_ 0.5 mM, and bFGF 5 ng/ml (Millipore, Billerica, MA) [20].

### 2.7. Transplantation of hMNPs ALS Model

*The SOD1 G93A* mutant mice were supplied and bred for ALS-TDI by GTC Biotherapeutics. Immune suppression was initiated 48 hours before procedures and consisted of a mixture of FK506/Rapamycin, 1 mg/kg of each. A laminectomy was performed at lumbar level L2 across two vertebral bodies at 60 days old. For transplantation, 32 G Hamilton needles with 30° bevels were used. A total of 40,000 hMNPs or CTS (vehicle) were injected intraspinally. Each injection was a total volume of 1 *μ*L, at a dose of 10,000 cells/*μ*L/injection. Animals were survived to 110 days old. 

Mice were divided into two groups of treated (transplantation with hMNPs; *n* = 25) and vehicle control (vehicle-injected; *n* = 25) groups that were evaluated up to the end stage for protein expression and histological evaluation of donor cell phenotype. Furthermore, 1 group of animals (composed of 15 mice for both hMNP treated and vehicle control SOD1 G93A mice) were analyzed for histological quantification and 1 group of animals were analyzed for molecular analysis (composed of 10 mice for both hMNP treated and vehicle control SOD1 G93A mice). 

*SMA Model. *At postnatal day 1, Smn^−/−^; SMNΔ7^+/+^; SMN2^+/+^ (hereafter referred to as Δ7SMN) pups were cryoanesthetized, and a laminectomy was performed in the upper lumber region. A glass-pulled tip attached to a Hamilton syringe was secured to a stereotactic apparatus and a total of 20,000 hMNPs (10,000 cell/uL) or volume equivalent of vehicle was transplanted bilaterally into the spinal cord [24]. Mice were divided into treated (transplantation with hMNPs; *n* = 11) and vehicle control (vehicle-injected; *n* = 4) groups that were evaluated up to the end stage for histological analysis. Animals were survived up to 14 days old following transplant.

*SCI Model. *Adult, female Sprague-Dawley rats (Charles River Laboratories, San Diego, CA) were anesthetized using Ketamine (100 mg/kg) and Xylazine (10 mg/kg, i.p.: Western Medical Supply, Los Angeles, CA) and underwent a T3 laminectomy. A 200 kD bilateral contusion injury was delivered using an Infinite Horizons Impactor (Precision Systems and Instruments, Lexington, KY). Animals received a solution of 5 mL ringers, 0.02 mL Baytril, and 0.4 mL diluted buprenorphine subcutaneously 2 days postinjury. Animals were also bladder expressed twice a day for minimum of 3 days, then as needed. Twenty-four hours prior to transplantation, animals began daily immunosuppression with cyclosporine A (20 mg/kg/d, s.c.; Bedford Laboratories, Bedford, OH). Animals also received antibiotic treatment of Baytril (0.02 mL) beginning one day prior to transplantation and continued throughout post-op. Seven days after spinal cord injury, animals received a transplantation of hMNPs; following reexposure of the laminectomy site, the spinal process of T2 was immobilized, and a 10 *μ*L Hamilton syringe (Hamilton, Reno, NV) with a 33 G beveled needle was lowered into the spinal cord using a stereotactic manipulator arm. A total of 100,000 hMNPs or CTS (vehicle) was injected in a total of four sites, 2 bilateral injections cranial and caudal to the injury site (0.5 mm lateral, 1.2 mm deep) to target the ventral horn. Each injection consisted of a total volume of 1 *μ*L, at a dose of 25,000 cells/*μ*L [20]. 

Mice were divided into two groups of treated (transplantation with hMNPs; *n* = 30) and vehicle control (vehicle-injected; *n* = 30) groups that were evaluated up to the end stage for protein expression and histological evaluation of donor cell phenotype. Furthermore, animals were split into 2 groups; the first group was (composed of 20 mice for both hMNP-treated and vehicle control SCI mice) analyzed for histological quantification and the second group of animals was analyzed for molecular analysis (composed of *n* = 10  mice for both hMNP-treated and vehicle control SCI mice). Animals were survived up to 3 months following transplant. 

### 2.8. Immunohistochemical Analyses of hMNPs

Animals were sacrificed via transcardiac perfusion with 4% paraformaldehyde. The tissue was cryopreserved in 27% sucrose, and the cords were sectioned into 1 mm blocks and embedded in OCT compound. Tissue was cryosectioned at 20 *μ*m and mounted onto gelatin-coated slides. Tissue sections were blocked with 10% NDS in PBS and permeabilized with 0.1% Triton X-100. To determine the fate of the transplanted hMNPs, sections were processed for multiple markers. Primary antibodies were added overnight at 4°C for Ku80 (1 : 300; Abcam), Islet-1 (1 : 200; Abcam), biotinylated NeuN (1 : 200; Millipore), p75 (1 : 200, Santa Cruz Biotechnology, Santa Cruz, CA), and ChAT (1 : 100; Millipore). Donkey antirabbit conjugated with biotin (1 : 250; Invitrogen), Streptavidin 594 (1 : 250; Invitrogen), Streptavidin 488 (1 : 250; Invitrogen), or goat antirabbit Alexa Fluor-488/-594 (1 : 250; Invitrogen) was used for 2 hours at room temperature as secondary antibodies.

### 2.9. Neuronal Sparing Analysis

The spinal cords from all three animal models were analyzed for neuronal sparing. Every tenth slide, spaced 200 *μ*m apart, was processed in order to visualize the length of the spinal cord [20]. IHC staining was performed on tissue slides using mouse anti-NeuN (1 : 200; Millipore) primary antibodies to identify neurons and rabbit anti-Ku80 (1 : 300; Abcam) primary antibodies to identify human nuclei. Serial sections were labeled with human nuclear antigen to ensure no human cells were included in the analysis. For motor neuron sparing analysis, mouse anti-ChAT (1 : 100; Millipore) primary antibodies and rabbit anti-Ku80 (1 : 300; Abcam) primary antibodies were used. The antibodies were diluted in PBS, incubated overnight at 4°C. Vector ABC Biotin Kit and Vectastain DAB Substrate Kits were used for secondary detection. Hoechst was used as a nuclear counterstain. Following IHC staining, the tissue was imaged using an Olympus AX-80 microscope. Images were taken of transverse sections of the ventral horns using MicroFire software. NeuN-positive neurons were quantified up to 2 mm cranial and 2 mm caudal to the injury epicenter or transplant site. The number of neurons was quantified using ImageJ software. Each image was opened individually and changed to an 8-bit image. Each image was then inverted, then the brightness and contrast was adjusted to the Auto setting, which made the gray areas darker and neurons were more easily visible. Then, threshold was adjusted from Default to MaxEntropy setting. Finally, the Nucleus Counter plug-in was used to detect pixels, the smallest particle size set to 400 and largest particle size set to 2000. Background subtraction and watershed filter were used in the plug-in. The plug-in then showed the black and white image of the neurons with a number indicating that it was counted and a summary table of the total counts. If a cell double stained positive for both NeuN and Ku80, then that cell was not used in quantification. Statistical analysis was performed using a Student's *t*-test (2 tailed distribution and two sample equal variance).

### 2.10. ELISA

Transplanted spinal cords were analyzed for growth factor expression using ELISA. Fresh epicenter blocks were flash frozen and then homogenized. Samples were homogenized in ice cold T-Per Protein Extraction Reagent (Pierce Biotechnology, Rockford, IL). Samples were kept at −20°C until analyzed with BCA protein assay. Triplicate supernatant samples were used to determine total protein levels present within the tissue using a BCA protein assay kit (Pierce, Rockford, IL). Optical densities were read on a THERMOmax ROM v1.72 plate reader at 450 nm, and the respective protein levels were quantified in comparison to a bovine serum albumin (BSA) standard curve. The ELISA kits used were NT-3 Emax Immuno Assay System (Promega), NGF Emax ImmunoAssay System (Promega), VEGF human ELISA kit (Invitrogen), NT-4 AccuBind ELISA kit (MonoBind, Inc.).

## 3. Results

### 3.1. In Vitro Differentiation

**Figure 1:**
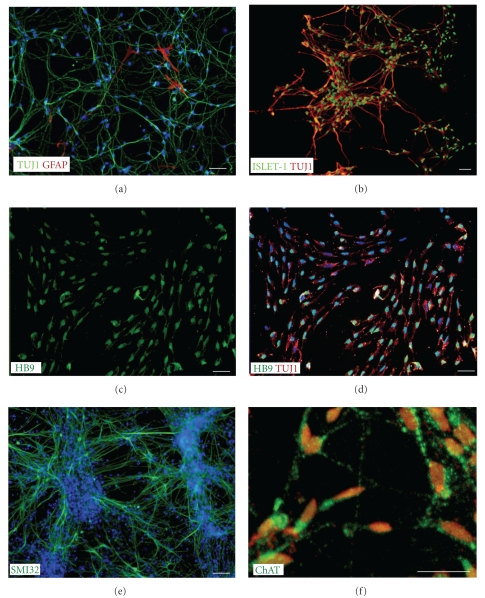
hMNP differentiation *in vitro*. At day 28, GFAP+ astrocytes ((a) red) were rare within TUJ1+ ((a) green) cultures. The majority of TUJ1+ cells ((b, d) red) double stained for the motor neuron lineage marker Islet-1 ((b) green) and HB9 ((c, d) green). After 3 weeks of subsequent growth, cells displayed a mature, branched morphology consistent with mature motor neurons, and expressed the mature motor neuron markers SMI-32 ((e) green) and ChAT ((f) green). Counterstain in blue (a, d, e) and red (f). Bar = 50 *μ*m.

At day 28, the day of transplantation, 93 ± 2.3% of cells expressed the neuronal axon marker TUJ1 (Figure 1(a)), 96 ± 1.7% of the cells expressed the motor neuron marker Islet-1 (Figure 1(b)), and 97 ± 2.6% of cells expressed the motor neuron marker HB9 (Figures 1(c) and 1(d)). Less than 1% of cells expressed the astrocyte marker GFAP (Figure 1(a)) and no cells expressed the mature motor neuron marker ChAT at day 28. No Oct4 positive stem cells could be identified on or after day 28 of the differentiation protocol. 

To assess maturation *in vitro*, hMNPs were plated on astrocytes and allowed to mature for a subsequent 3 weeks following day 28 of the differentiation protocol (Figures 1(e) and 1(f)). Astrocytes did not immunostain for the motor neuron markers SMI-32 (Figure 1(e)) and ChAT (Figure 1(f)), but were evident by hoescht staining. Quantification of astrocyte positive cells was therefore not performed. The neurofilament marker SMI-32 (Figure 1(e)) was expressed by 97.7 ± 1.53% of the cells and 96.6 ± 4.4% of the cells expressed the mature motor neuron marker ChAT (Figure 1(f)); while HB9 expression decreased significantly (*P* < .001; 6 ± 4.4%) from the day 28 differentiation profile. Motor neuron maturation was further demonstrated by kainate-stimulated uptake of Co^2+^ ions by Ca^2+^ permeable AMPA channels (not shown).

### 3.2. In Vivo Differentiation

**Figure 2:**
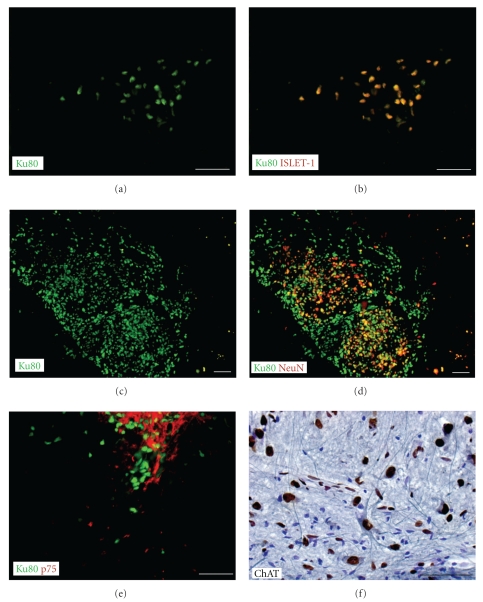
hMNP differentiation *in vivo*. Human nuclear (Ku80) antigen-positive cells were detected in all treated animals of all 3 animal models and colabeled with different motor neuron differentiation markers. Human nuclear (Ku80) antigen-positive cells (a, c) double stained with Islet-1 ((b) red), NeuN ((d) NeuN in red, human nuclei in green), p75 ((e) p75 in red, human nuclei in green), or ChAT ((f) ChAT in blue, human nuclei in brown), consistent with a motor neuron lineage of mixed maturation state. Bar = 50 *μ*m.

Human nuclear (Ku80) antigen-positive cells were detected in all transplanted animals of all 3 animal models and did not migrate from the transplant sites cranial and caudal to the injection sites (Figure (2)). Human cells (Figures 2(a) and 2(c)) double stained with the young motor neuron marker Islet-1 (Figure 2(b)) and were observed in the ventral horns of hMNP treated but not in vehicle control Δ7SMN animals. In Δ7SMN animals, human nuclear antigen-positive cells did not double label with markers for the mature motor neuron markers ChAT or SMI-32, indicating that their limited time *in vivo* (13 days) is insufficient for differentiation of transplanted hMNPs. However, human cells were found within the SOD1 G93A and SCI animals, in which animals were survived for up to 2 and 3 months, respectively, after transplantation. Human cells were colabeled with neuronal and motor neuron markers such as NeuN (Figure 2(d)), p75 (Figure 2(e)) and ChAT (Figure 2(f)), consistent with a motor neuron lineage of a mixed maturation state. Many human nuclear antigen-positive cells extended TUJ1-positive processes. TUJ1 tissue staining was absent in no-primary and no-secondary antibody controls. Transplanted cells did not extend axons into the periphery or form neuromuscular junctions with host tissue, as expected.

### 3.3. In Vitro Growth Factor Secretion

**Figure 3:**
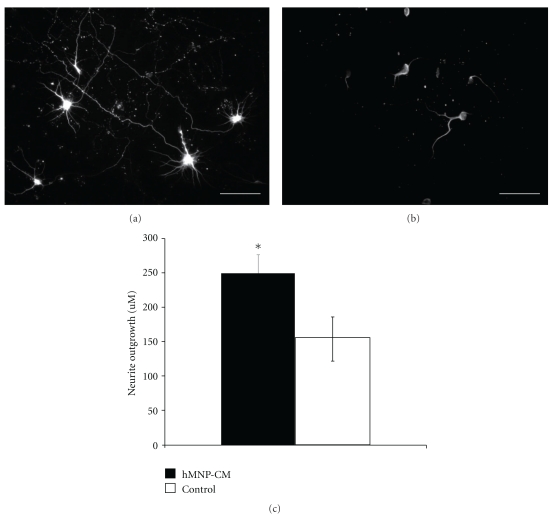
hMNPs cause neurite branching *in vitro*. Neurite length was significantly longer (*P* < .05) in cortical neuron cultures exposed to hMNP-conditioned media (a) for 7 days as compared to cortical neuron cultures exposed to control MN differentiation media (b). (c) The length of neurites in cultures exposed to hMNPconditioned media as compared to those exposed to control media was significantly higher (*P* < .05). Bar = 50 *μ*m.

Rossi et al [20] have previously shown that hMNP's secrete various growth factors. To investigate the neurotrophic potential of hMNP secretions, we performed functional assays of hMNP secretions on cortical neurons *in vitro *(Figure 3). Neurite length of cortical neuron cultures exposed to hMNP-conditioned media (Figure 3(a)) was compared to neurite length of cortical neuron cultures exposed to control MN differentiation media only (without conditioning) (Figure 3(b)) and was found to be significantly greater (*P* < .05; (Figure 3(c)). Neurite length of cortical neuron cultures exposed to hMNP-conditioned media was 250 *μ*m ± 27 *μ*m, and neurite length of cortical neuron cultures exposed to MN differentiation media only was 151 *μ*m ± 32 *μ*m.

### 3.4. In Vivo Growth Factor Secretion

**Figure 4:**
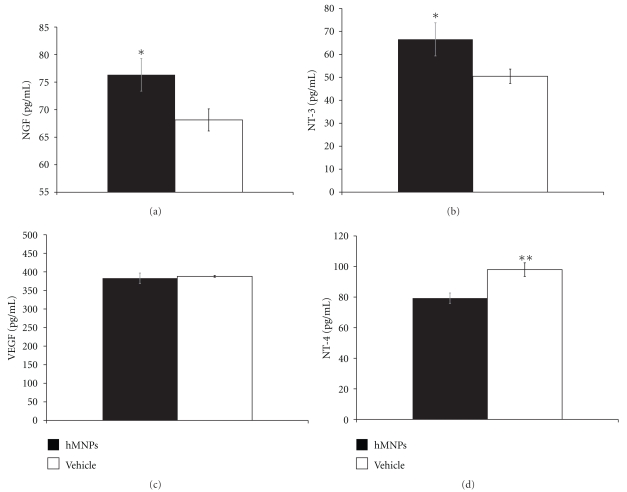
hMNPs secrete physiologically active growth factors *in vivo*. ELISA data of NGF (a), NT-3 (b), VEGF (c), and NT-4 (d) protein levels in the thoracic spinal cord transplanted region of SCI rats. Data is expressed as mean ± standard error. *P < .05; **P < .01.  *n* = 6 hMNP; *n* = 9 vehicle.

The presence of growth factors NGF, NT-3, VEGF, and NT-4, in the spinal cord of animals was assessed in whole protein extracts via ELISA (Figure 4). The levels of NGF protein were significantly greater (*P* < .05) in hMNP-transplanted animals (76.3 ± 3.0 pg/mL) as compared to vehicle control animals (68.1 ± 2.0 pg/mL; Figure 4(a)). Similarly, the levels of NT-3 protein were significantly greater (*P* < .05) in hMNP-transplanted animals (66.5 ± 7.2 pg/mL) as compared to vehicle control animals (50.5 ± 3.1 pg/mL; Figure 4(b)). There was no significant difference (*P* > .05) in the level of VEGF between the vehicle control (387.8 ± 13.3 pg/mL) and hMNP transplanted animals (382.9 ± 13.9 pg/mL; Figure 4(c)). Interestingly, NT-4 levels were significantly greater (*P* < .01) in the vehicle control animals (98.0 ± 4.5 *μ*g/mL), as compared to hMNP-transplanted animals (79.3 ± 3.4 *μ*g/mL; Figure 4(d)).

### 3.5. Neuronal Sparing

**Figure 5:**
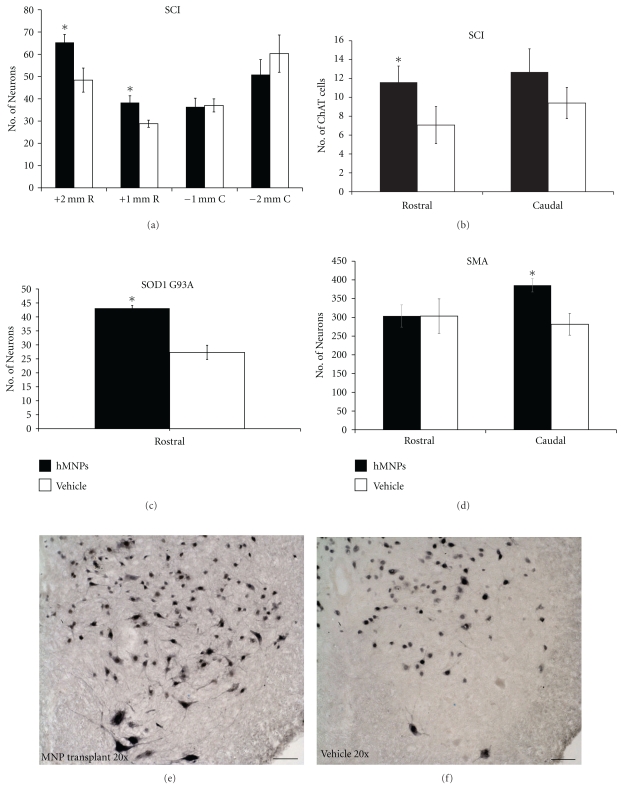
hMNP transplantation spares endogenous neurons. (a) Comparison of NeuN positive cells in cranial (*n* = 13 hMNP; *n* = 15 vehicle) and caudal (*n* = 14 hMNP; *n* = 15 vehicle) transverse sections in SCI animals. (b) Comparison of ChAT positive cells in cranial (*n* = 10 hMNP; *n* = 12 vehicle) and caudal (*n* = 9 hMNP; *n* = 12 vehicle) transverse sections of ventral horns in SCI animals. (c) Comparison of NeuN positive cells in cranial transverse sections of SOD1 G93A animals (*n* = 5 hMNP; *n* = 5 vehicle). (d) Comparison of NeuN positive cells in cranial (*n* = 3 hMNP; *n* = 3 vehicle) and caudal (*n* = 3 hMNP; *n* = 2 vehicle) transverse sections of Δ7SMN animals. NeuN stained histological sections of lumbar spinal cord from hMNP (e) and vehicle (f) injected into SOD1 G93A mice clearly show a difference in neuronal numbers. Data is expressed as mean ± standard error. *P < .05. Bar = 50 *μ*m.

The average number of neurons in the hMNP treated group and vehicle control group of all 3 animals was assessed for endogenous neuronal sparing (Figure 5). In SCI and SOD1 G93A hMNP-treated animals cranial to the injury/injection epicenter, there were 38 ± 3 and 43 ± 5 endogenous neurons counted, respectively. The average number of neurons in the vehicle control group of SCI and SOD1 G93A animals cranial to injury/injection epicenter was 29 ± 2 and 27 ± 3, respectively. Cranial to the injury/injection epicenter, the hMNP-treated group showed a significant sparing of neurons compared to the vehicle control group in SCI (Figure 5(a); *P* < .01) and SOD1 G93A animals (Figure 5(c); *P* < .05). The average number of neurons in the hMNP-treated group of SCI animals caudal to the injury/injection epicenter was 36 ± 4. The average number of neurons in the vehicle control group of SCI animals caudal to the injury/injection epicenter was 37 ± 3. The hMNP-treated group did not demonstrate statistical significance in neuronal sparing in SCI or SOD1 G93A animals caudal to the injury/injection epicenter. Therefore, caudal to the injury/injection epicenter, neuronal sparing was not affected by the transplantation of hMNPs, whereas hMNP transplantation (Figure 5(e)) did spare neurons cranial to the injury/injection epicenter and the vehicle control did not (Figure 5(f)).

The average number of neurons in the hMNP treated group in Δ7SMN animals cranial to the injection epicenter was 304 ± 30. In the vehicle control group, the average number of neurons cranial to injection epicenter was 304 ± 46. Cranial to the injection epicenter, the hMNP-treated group did not demonstrate a significant sparing of neurons compared to the control group (Figure 5(d)). The average number of neurons in the hMNP-treated group in Δ7SMN animals caudal to the injection epicenter was 386 ± 18. However, average number of neurons in the vehicle control group caudal to the injection epicenter was 282 ± 29. Caudal to the injection epicenter, the hMNP-treated group showed statistical significance in neuronal sparing (Figure 5(d); *P* < .05). Therefore, cranial to the injection epicenter, neuronal sparing was not affected by the transplantation of hMNPs, whereas hMNP transplantation did spare neurons caudal to the injection epicenter.

### 3.6. Motor Neuron Sparing

The average number of ChAT positive cells in the hMNP treated and vehicle control groups was compared to examine whether neuronal sparing may have included endogenous motor neurons (Figure 5(b)). The average number of ChAT-positive cells within the ventral horn in SCI animals of the hMNP-transplanted group cranial to the injury/injection epicenter was 12 ± 2. The average number of ChAT positive cells within the ventral horn in SCI animals of the vehicle control group cranial to the injury/injection epicenter was 7 ± 2. The number of ChATpositive cells cranial to the injury/injection epicenter in the hMNP treated group was significantly greater (*P* < .05) than that of the vehicle control group, demonstrating that hMNP transplantation resulted also in the specific sparing of endogenous motor neurons. There was no significant difference in the number of ChAT positive cells caudal to the injury/injection epicenter, although there is a trend towards increased sparing. The number of ChAT-positive cells caudal to the injury/injection epicenter in the hMNP treated group was 13 ± 2. The number of ChAT-positive cells caudal to the injury/injection epicenter in the vehicle control group was 9 ± 2.

## 4. Discussion

Transplantation is an inherently combinatorial therapeutic approach, as it may provide neurotrophic support for the endogenous environment, and cell phenotype-related benefits such as replacement of dead or dying cells, a substrate for endogenous growth, or a tissue bridge for endogenous growth. The relative contribution of neurotrophic support and phenotypic benefits conferred by a transplant is difficult or impossible to discern for transplants that consist of mixed lineages. In this study, we investigated the neurotrophic effects of a high purity motor neuron lineage graft in three animal models of motor neuron loss. We demonstrate that hMNPs secreted physiologically active growth factors *in vitro* and *in vivo* and significantly enhanced the number of spared endogenous neurons following transplantation in three animal models of motor neuron loss. The ability to maintain dying motor neurons by delivering motor neuron-specific neurotrophic support represents a powerful treatment strategy for diseases characterized by motor neuron loss.

Motor neurons respond to neurotrophic cues and express and secrete various growth factors [25]. Our data indicate that hMNPs secrete NT-3, and NGF, and our previous publication further indicates that they secrete VEGF and NT-4 [20]. NT-3 and VEGF have known beneficial effects on CNS pathogenesis [16, 26]. Furthermore, our *in vitro *data demonstrate that hMNP-conditioned media enhanced elongation and branching of cortical neurites, demonstrating that the growth factor secretions from hMNPs were physiologically relevant. 

Our transplantation studies further confirm that hMNPs have physiologically relevant neurotrophic effects in the vicinity of the transplant. hMNP transplantation into SMA, ALS, and SCI animal models resulted in beneficial effects on injury pathogenesis, as evidenced by a greater number of spared endogenous neurons in all three animal models. Although the growth factors secreted by hMNPs *in vivo *have been implicated in neuronal survival [16, 26], the mechanisms underlying their interaction with the endogenous environment are unknown. Their mechanism of action may be direct, or indirect, as trophic support of glial cells and/or modulation of the immune or glial response may have contributed to survival of endogenous neurons. Neurons within regions of pathology upregulate p75 neurotrophin receptors (p75NTR) and downregulate tyrosine-related kinase receptors (TrkRs) [16, 26]. The p75NTR binds the precursor forms of neurotrophins and has been implicated in apoptosis, while TrkRs bind mature forms of neurotrophins and have been implicated in beneficial neurotrophic effects [16, 26]. hMNP-secreted growth factors may prevent, inhibit, or counteract neurotrophic action at the p75NTR and/or enhance activation and/or upregulation of TrkRs, enhancing downstream neurotrophic effects. 

This study directly assessed the potential of a cell-based therapeutic to confer neurotrophic benefit to regions of pathogenesis in the absence of phenotypic benefit. hMNP transplants did not extend processes to reinnervate distant muscles, so their neurotrophic benefit to the diseased or injured spinal cord could be investigated in isolation from phenotypic benefits conferred by a transplant. These findings further our understanding of the neurotrophic benefits of hMNP transplantation. Nonetheless, efforts to mature transplanted hMNPs and enhance axonal projection to target muscles are important to maximize the benefits of this cell population.
